# Transcriptional Activity of rRNA Genes in Barley Cells after Mutagenic Treatment

**DOI:** 10.1371/journal.pone.0156865

**Published:** 2016-06-03

**Authors:** Jolanta Kwasniewska, Joanna Jaskowiak

**Affiliations:** Department of Plant Anatomy and Cytology, Faculty of Biology and Environmental Protection, University of Silesia in Katowice, Katowice, Poland; Laval University Cancer Research Centre, CANADA

## Abstract

In the present study, the combination of the micronucleus test with analysis of the activity of the rRNA genes in mutagen-treated *Hordeum vulgare* (barley) by maleic hydrazide (MH) cells was performed. Simultaneously fluorescence *in situ* hybridization (FISH) with 25S rDNA as probes and an analysis of the transcriptional activity of 35S rRNA genes with silver staining were performed. The results showed that transcriptional activity is always maintained in the micronuclei although they are eliminated during the next cell cycle. The analysis of the transcriptional activity was extended to barley nuclei. MH influenced the fusion of the nucleoli in barley nuclei. The silver staining enabled detection of the nuclear bodies which arose after MH treatment. The results confirmed the usefulness of cytogenetic techniques in the characterization of micronuclei. Similar analyses can be now extended to other abiotic stresses to study the response of plant cells to the environment.

## Introduction

Plant bioassays are commonly used to detect chromosome rearrangements induced by environmental, possibly mutagenic, factors. The application of modern cytogenetic techniques such as fluorescent *in situ* hybridization (FISH) permits more detailed analyses of chromosome aberrations and micronuclei. A cytogenetic study of physical location of 5S rRNA genes and genes encoding for 18S, 5.8S and 25S rRNAs (35S rRNA), in a few *Hordeum* species proved that rDNA provides useful chromosome landmarks [1]. A micronucleus (MN) test combined with FISH allowed a quantitative analysis of the involvement of specific chromosome fragments in micronuclei formation and thus permitted the possible origin of mutagen-induced micronuclei to be explained. 35S rDNA are genes that belong to the category of “housekeeping genes”, which are present in every living cell and they have to have at least basal activity. In a previous study an attempt was made to identify the origin of micronuclei using FISH with rDNA as probes. A quantitative analysis of the involvement of specific chromosome fragments in micronuclei in barley cells was done using FISH. The characterization of micronuclei by identifying the rRNA genes involved in their formation in barley cells showed that rDNA-bearing chromosomes are often involved in micronuclei formation [2,3]. The commonly used histochemical silver staining, which is used to detect active ribosomal genes, can be useful in the analysis of the activity of the 35S rRNA genes in the micronuclei. Although the reuse of slides to localize rRNA genes and analyze the transcriptional activity of these genes has found wide application in plant cytogenetics [4,5], it has not yet been used in mutagenesis.

In this study, analyses of localization of 35S rRNA using FISH and the transcriptional activity of 35S rRNA genes with silver staining, were performed simultaneously. It permitted a determination whether the presence of rRNA genes in micronuclei is coupled with their activity. Maleic hydrazide (MH), which is a commonly known clastogenic agent that can lead to chromosome breaks as well as cause spindle fiber defects [2], was used as the model mutagen. It is worth investigating the activity of rRNA genes in micronuclei because they are eliminated during the next cell cycle. Additionally, an analysis of the influence of MH on the number of nucleoli and nuclear bodies in barley cells was applied.

## Material and Methods

### Material and treatment

Barley (*Hordeum vulgare*, 2n = 14) seeds of the “Start” variety were presoaked in distilled water for 8 h and then treated with maleic hydrazide − 3 mM or 4 mM (MH; Sigma, CAS 123–3301) for 3 h. After treatment the seeds were washed three times in distilled water and then germinated in Petri dishes on wet filter paper at 21°C. The material was fixed in ethanol: glacial acetic acid (3:1) 72 h after treatment. The roots of M_1_ seedlings were used as the source of the meristems for the analyses.

### Chromosomal preparations

For chromosome preparations the material was washed with a 0.01 mM sodium citrate buffer (pH 4.8) for 30 min and digested with an enzyme mixture containing 2% cellulase (w/v, Onozuka, Serva) and 20% pectinase (v/v, Sigma) for 2 h at 37°C. After digestion the material was washed again with a sodium citrate buffer for 30 min. Squash preparations were made in a drop of 45% acetic acid. After freezing and removing coverslips, the slides were dried.

### Silver staining

Silver staining was done using the modified method of Hizume et al. [6]. Prior to staining slides were treated with a borate buffer (pH 9.2; Merck) for 30 min. Then, a few drops of freshly prepared 50% AgNO_3_ (Merck) in distilled water were applied to each preparation. Slides were covered with a nylon mesh and incubated in a humid chamber at 42°C for 70 min, washed in distilled water and air-dried. The photographs were taken with a Zeiss Axio Imager.Z.2 wide-field microscope equipped with an AxioCam Mrm monochromatic camera. The images were captured and processed using Adobe Photoshop 4.0.

After analysis of the silver-stained preparations, the immersion oil was removed from the preparations with 100% acetone (Poch). The slides were rinsed briefly in 4xSSC (0.3 M NaCl, 0.03 M sodium citrate) at 37°C to remove the coverslips and then for 5 min in 4xSSC at 37°C. To remove the silver staining, the preparations were placed in 30% hydrogen peroxide (Poch) for 20–30 s, rinsed 3 x 5 min in distilled water and air-dried. The slides were fixed in ethanol: glacial acetic acid (3:1) for 10 min and then 100% ethanol for 5 min. The slides were air-dried and then used for *in situ* hybridization.

Fluorescence *in situ* hybridization (FISH)

Fluorescence *in situ* hybridization was applied according to the method described by Maluszynska and Heslop-Harisson [7] with some minor modifications. 25S rDNA, which had been isolated from *Arabidopsis thaliana* [8] and labeled with digoxygenin-11-dUTP using nick translation (Roche), was used as the probe.

Prior to FISH, the chromosome preparations were pretreated with RNase (1 mg/ml) for 60 min at 37°C and washed 3 times for 5 min in 2 x SSC at room temperature, dehydrated in methanol series and air-dried.

The hybridization mixture containing 2.5 μg/ ml of labeled DNA, 50% (v/v) formamide, 10% (w/v) dextran sulphate and 0.1 mg/μl salmon testes DNA in 2 x SSC was denaturated at 75°C for 10 min and immediately placed on ice for a few min. The hybridization mixture (38 μl) was added to the chromosome preparations and covered with a plastic coverslip. The chromosomes and DNA probes were denatured for 5 min at 70°C on a hot plate (Hybaid Thermal Cycler PCR). Hybridization was carried out in a moist chamber at 37°C for 20 h. After hybridization, the slides were washed for 4 min in 2 x SSC at 42°C, 2 x 4 min in 0.1 SSC at 42°C, 3 x 3 min in 2 x SSC at 42°C, 3 x 3 min in 2 x SSC at room temperature and in 0.2% Tween in 4 x SSC at room temperature for 5 min.

The digoxigenin-labeled probe was detected using FITC-conjugated anti-digoxigenin antibodies (Roche) and then the signal was amplified using a FITC-conjugated secondary antibody (FITC-conjugated Anti-Sheep; Dako). After three washes for 8 min in 0.2% Tween in 4 x SSC at 37°C and dehydration in an ethanol series, the slides were mounted in a Vectashield medium (Vector) containing 6 μg/ ml DAPI.

Preparations were examined with a Zeiss Axio Imager.Z.2 wide-field fluorescence microscope equipped with an AxioCam Mrm monochromatic camera. The images were captured and processed using Adobe Photoshop 4.0.

The frequency of micronuclei with specific DNA signals and without signals was calculated. For each experimental group, 50 cells with micronuclei on three slides, each made from three meristems, were evaluated. The results of the analyses were pooled for all of the concentrations of MH. The total frequency of micronuclei was estimated on the same slides − 2000 cells in the control and the treated material were analyzed. The data on frequencies of nuclei were analyzed using a nonparametric Kruskal-Wallis one-way analysis of variance by rank test. If a significant K-value of P<0.05 was obtained, a Mann-Whitney U test was performed.

## Results and Discussion

**Fig 1:**
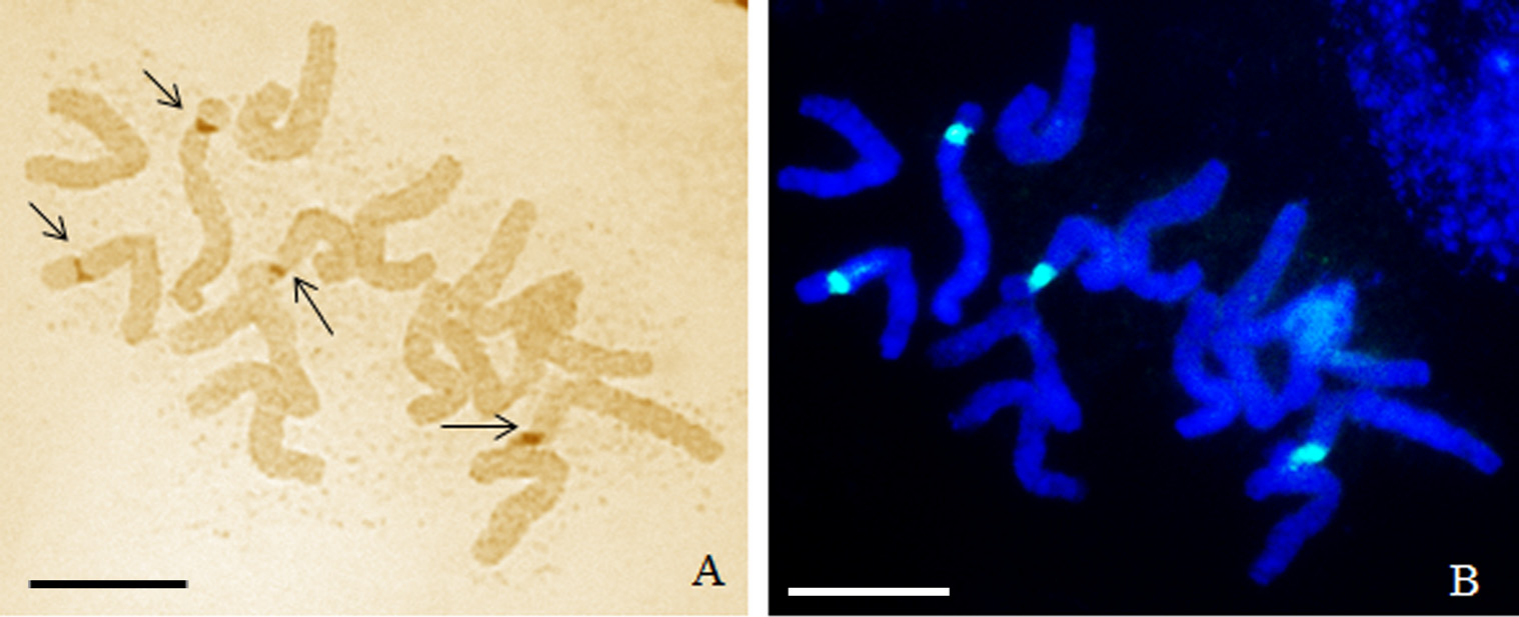
The metaphase chromosomes of *H*. *vulgare* Start var. (2n = 14). (A) Silver-staining method showing the active loci of 35S rDNA (B) fluorescence *in situ* hybridization showing the distribution of all 35S rRNA genes (right). Bars represent 10 μm

**Fig 2:**
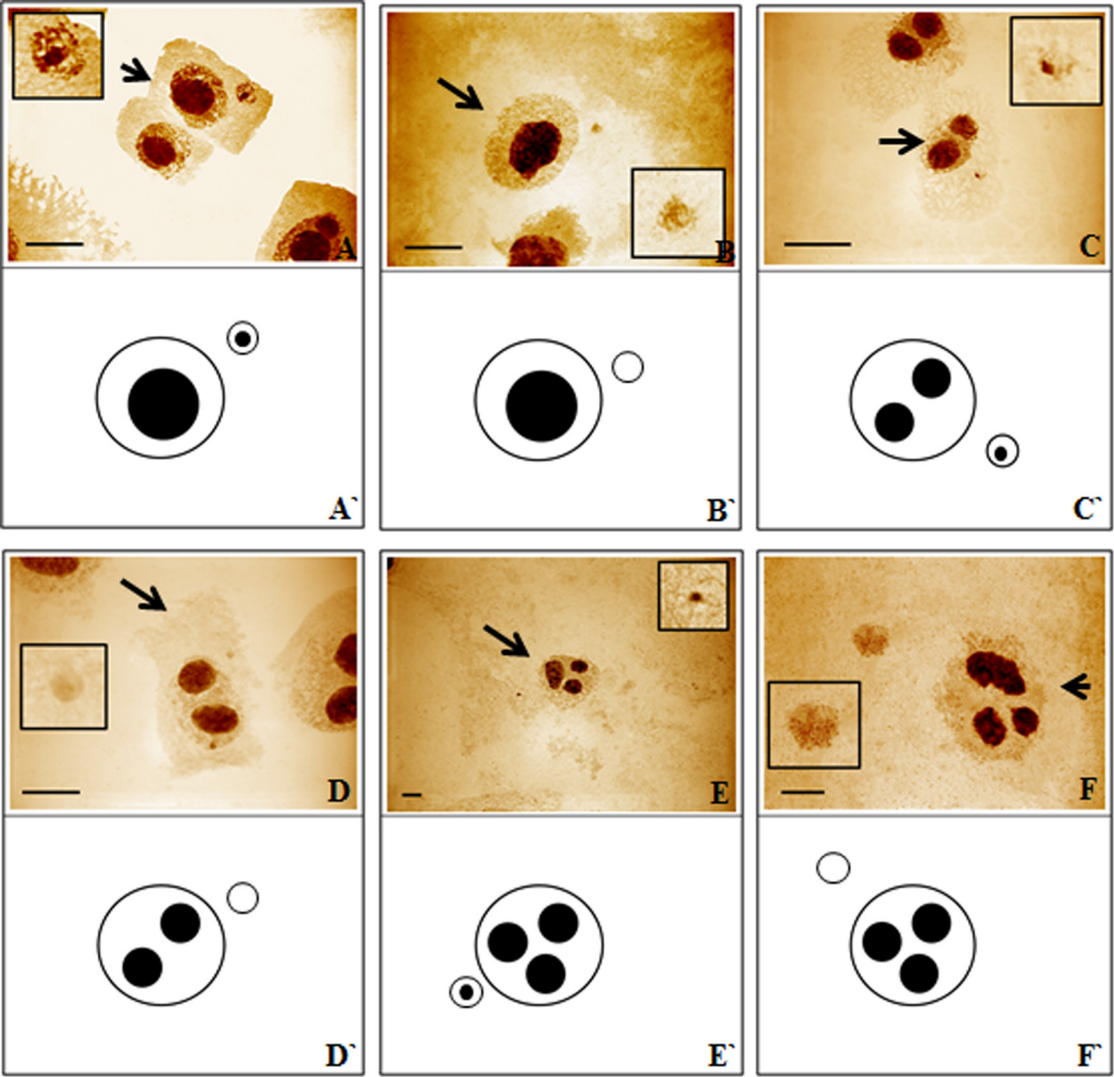
*H*. *vulgare* root meristematic cells with micronuclei after treatment with MH–silver-staining method. **(A-F) original images, arrows indicate cells chosen for drawings. Images of micronuclei are enlarged. (A`-F`) model drawings based on orginal images.** Bars represent 10 μm.

**Fig 3:**
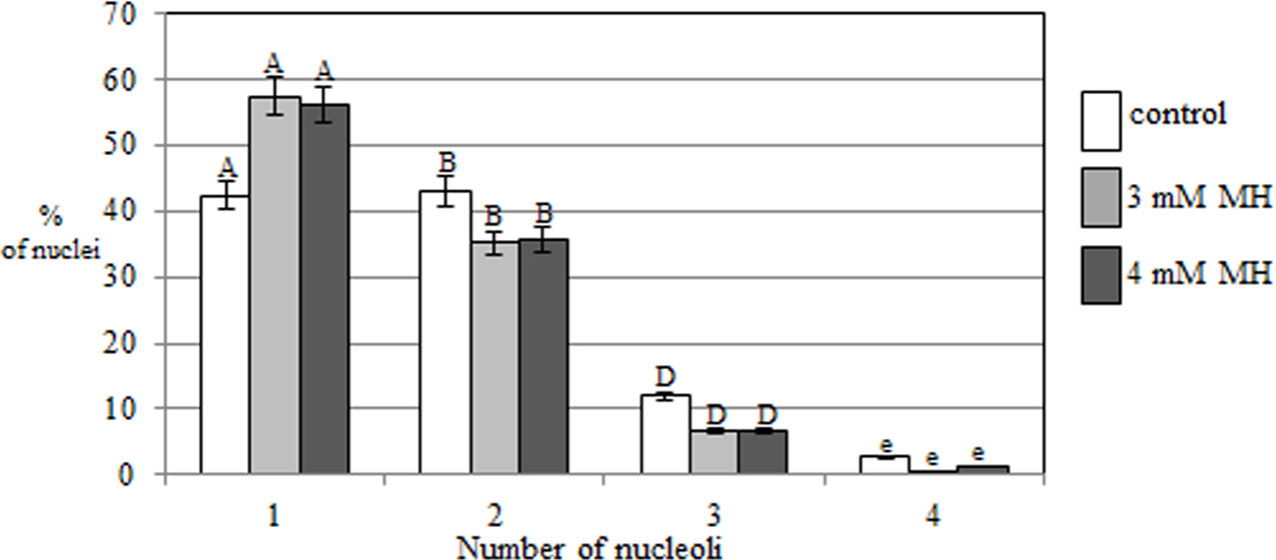
The frequencies of *H*. *vulgare* nuclei with a different number of nucleoli (from 1 to 4) in root meristematic cells in the control and after MH treatment. The bars represent standard errors (SE). The capital letters A, B, D indicate that the frequencies of nuclei with 1, 2 or 3 nucleoli are significantly different (P<0.05) between the each of the experimental group: control, 3 mM MH and 4 mM MH. The lower case letter e indicates that the frequencies of nuclei with four nucleoli are not significantly different (P<0.05) between the control, 3 mM MH and 4 mM MH.

In the present study, the activity of the 35S rRNA genes in MH-treated *H*. *vulgare* (2n = 14) cells was evaluated using the silver-staining method and fluorescence *in situ* hybridization. *H*. *vulgare* is a very good model for such investigations due to its convenient karyotype features—large chromosomes, two of which display the presence of 35S rRNA genes, together with activity of all of them are convenient karyotype features that made barley a suitable experimental model for this study (Fig 1). The transcriptional activities of the all of the 35S rRNA gene loci in the barley cells permitted an analysis of their activities in the micronuclei, which are eliminated from the cells in the next cell cycle. The use of the silver-staining method permitted an analysis of the activity of 35S rRNA genes in micronuclei that were induced by MH-treatment (Fig 2). The presence of the signals from histochemical staining in a micronucleus indicates that this micronucleus originated from the 35S rDNA region or the entire chromosome/chromosome arm and this confirmed the activity of these genes. The lack of signals in micronuclei could indicate the absence of 35S rRNA genes or their activity. The presence or lack of silver-staining signals in micronuclei was not correlated with the number of nucleoli or the size of micronuclei (Fig 2). The number of nucleoli in the *H*. *vulgare* Start var. control cell was one to four and correlated with two pairs of NOR chromosomes, which were transcriptionally active. However, the observed number of nucleoli is generally lower than the number of active rDNA loci due to their fusion during the cell cycle [9]. Treatment with MH influenced the number of nucleoli in the barley cells (Fig 3). The frequency of nuclei with one nucleolus increased significantly after treatment with MH–from 42.3% in the control to about 57% in the MH-treated cells. At the same time, the frequencies of the nuclei with two or three nucleoli were significantly lower after MH treatment in comparison with the control. The results of this study clearly indicated that MH could influence the fusion of the nucleoli in barley cells. This process occurred before the G1 of the cell cycle [10]. MH is responsible for disturbances in the biosynthesis of nucleic acids and proteins [11]. The nucleolus is very sensitive to stressful factors. The frequency of cells with one nucleus increases in response to heavy metals or changes of temperature [9]. In this study we have shown the influence of the MH on the number of nucleoli in plant cells for the first time. An increased size of the nucleoli was observed in *A*. *cepa* after treatment with another inhibitor of protein biosynthesis in eukaryotic cells by cycloheximide [12].

**Fig 4:**
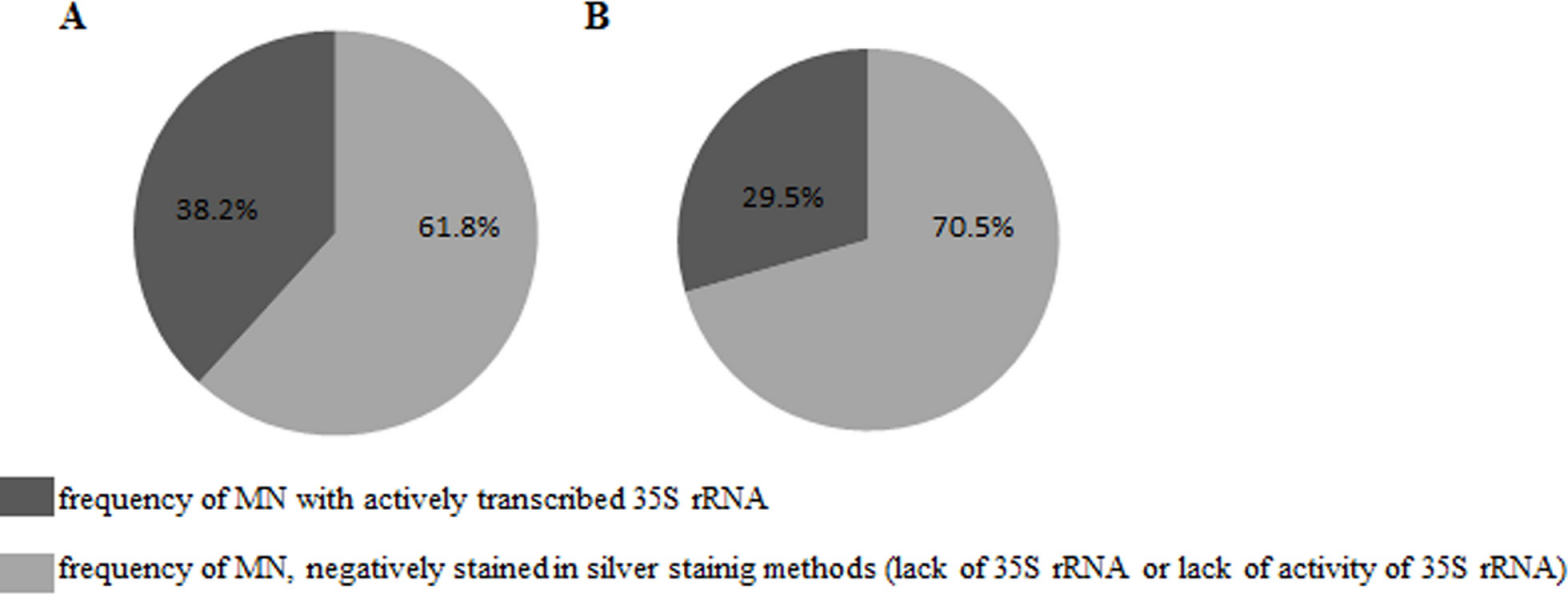
Frequencies of micronuclei with silver-staining signals (indicating the presence of active rRNA genes) or without silver-staining signals (indicating a complete lack of rRNA genes or a lack of active rRNA genes). (A) 3 mM MH, (B) 4 mM MH

**Fig 5:**
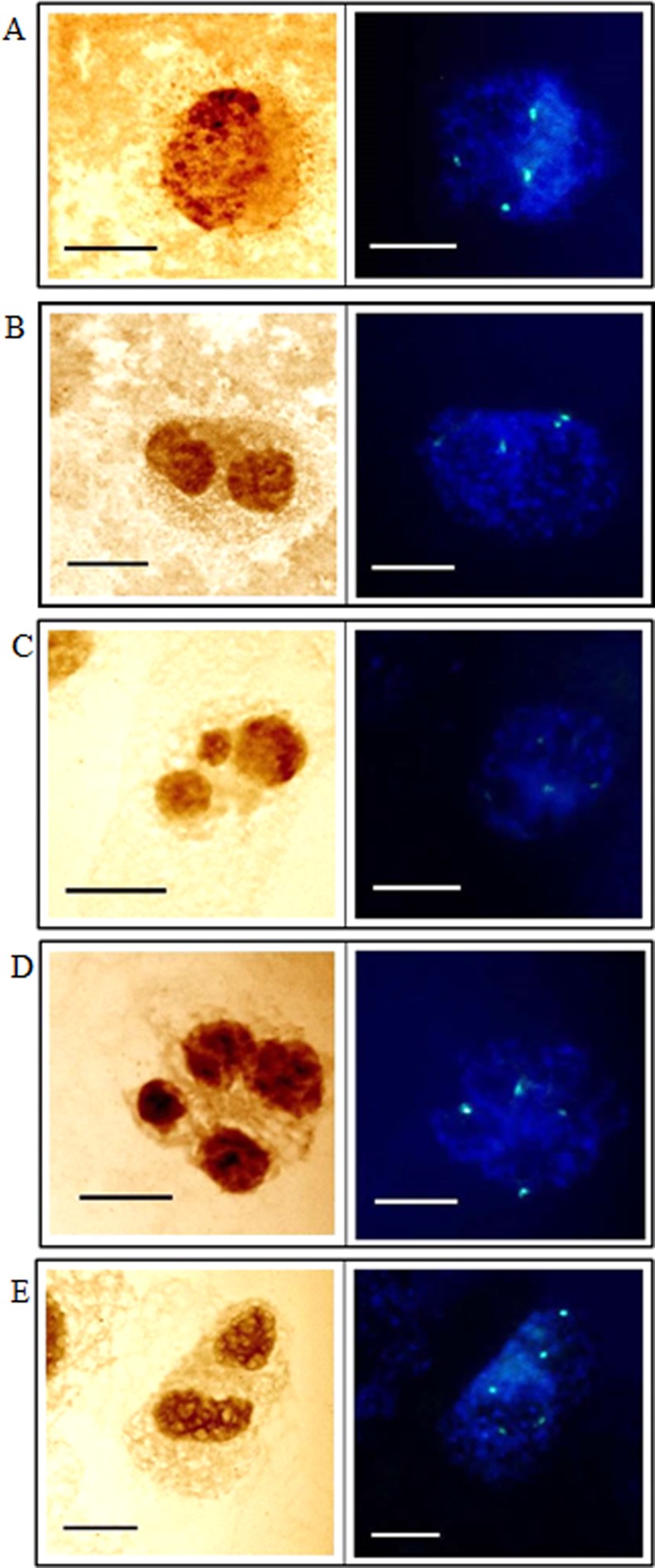
Untreated *H*. *vulgare* interphase cells without micronuclei. **Staining with the silver-staining method (left columns) and fluorescence *in situ* hybridization with 25S rDNA as the probe (right columns) applied to the same cells.** Bars represent 10 μm

**Fig 6:**
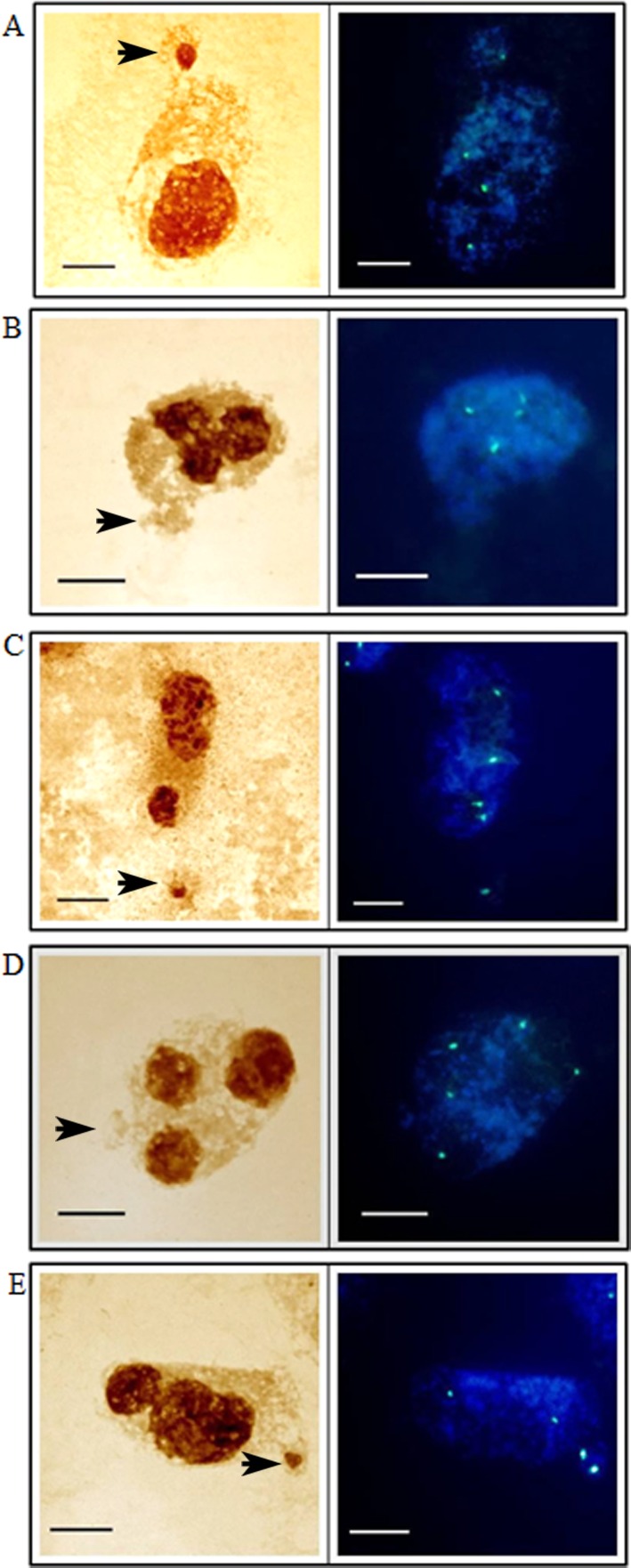
*H*. *vulgare* interphase cells with micronuclei after treatment with MH. Staining with the silver staining-method (left columns) and fluorescence *in situ* hybridization with 25S rDNA as the probe (right columns) applied to the same cells. Bars represent 10 μm.

An analysis of the frequencies of the micronuclei with silver-staining signals was then conducted. The frequencies of micronuclei with signals that indicated the presence of active 35S rRNA genes were dependent on the MH concentration– 38.2% after treatment with 3 mM MH and 29.5% after treatment with 4 mM MH (Fig 4). FISH with 25S rDNA as probe was used in the next experiments because the use of silver staining did not show the proportion of micronuclei with active 35S rRNA genes to the micronuclei that had these genes. Before the analysis of the micronuclei, an evaluation of the number of 35S rDNA signals using FISH together with an analysis of the number of nucleoli in the control cells without micronuclei were applied (Fig 5). Generally, four foci of 35S rDNA were observed in the nuclei (Fig 5A–5D). The number of 35S rDNA signals did not correlate with the number of nucleoli. Sporadically, the number of 35S rDNA signals in the nuclei was five (Fig 5E). The presence of five signals of 35S rDNA can indicate the probability of a duplication event including this region or of a break inside it. The sequential use of the silver-staining method and then fluorescence *in situ* hybridization with 25S rDNA as probe to the MH-treated root meristematic cells of barley with micronuclei permitted the simultaneous analysis of the presence of 35S rRNA genes and their activity (Fig 6). The number of 35S rDNA signals in the nuclei from the cells with micronuclei ranged from two to five. Nuclei with three signals of 35S rDNA and one signal in the micronucleus (Fig 6A) or nuclei with four signals but without any signals in the micronucleus (Fig 6B) were observed most often. Sporadically, nuclei with four signals of 35S rDNA and one signal in the micronucleus (Fig 6C) and nuclei with five signals and the absence of a signal in the micronucleus (Fig 6D) were also observed. However, cells with two FISH signals in the nucleus and two signals in the micronucleus were rarely observed (Fig 6E). Summarizing, all of the 35S rDNA foci that were present in the micronuclei were transcriptionally active. All of the 35S rDNA loci are active in *H*. *vulgare* Start var. cells, and therefore this result is predictable. However, it was interesting to determine whether this activity is maintained taking into account the fact that micronuclei are eliminated during the next cell cycle.

**Fig 7:**
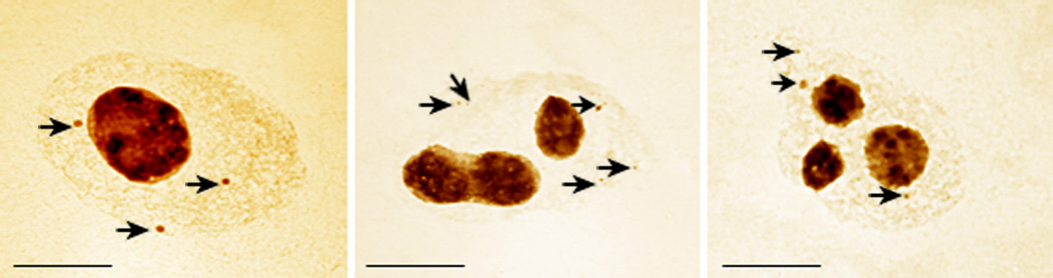
*H*. *vulgare* nuclei with nuclear bodies after treatment with MH–silver-staining method. Bars represent 10 μm.

**Fig 8:**
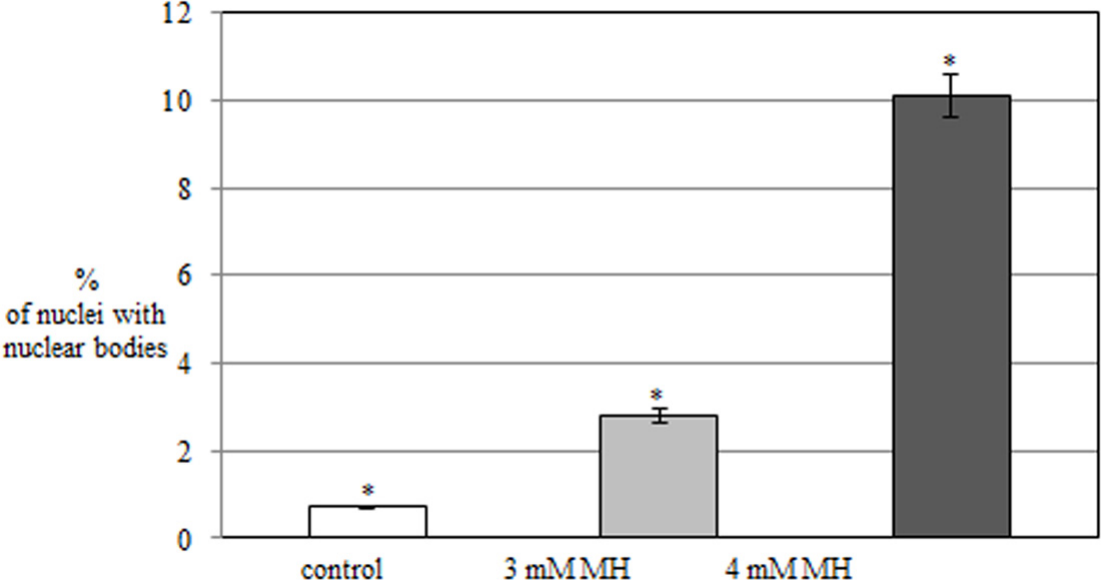
The frequencies of *H*. *vulgare* nuclei with nuclear bodies in the control and MH-treated cells. The bars indicate standard errors (SE). An * indicates that the frequencies of nuclei with nuclear bodies are significantly different (P<0.05) between the experimental groups.

An analysis of the silver-stained cells treated with MH showed the presence of nuclear bodies. They were small, positively labeled structures (Fig 7). The size of the nuclear bodies was much smaller than the micronuclei. The number of nuclear bodies in the barley cells varied and ranged from one to three. Neither the presence nor the number of nuclear bodies correlated with the number of nucleoli (data not shown). Nuclear bodies were sporadically observed in the control cells (0.7%). Their frequency significantly increased after MH treatment and correlated with the concentration of the mutagen. Only 2.8% of cells that were treated with 3 mM MH and 10.1% of cells treated with 4 mM MH had nuclear bodies (Fig 8). Nuclear bodies have not been previously observed in any of the dividing or slowly dividing root cells of non-treated cells of *A*. *thaliana*, *B*. *napus*, *P*. *sativum* or *Z*. *mays* [13, 14]. Aluminum and lead are known as factors that induce nuclear bodies [15–17]. Although nuclear bodies have been observed in species that belong to grasses, e.g. *Oryza sativa* and *Tritcum aestivum* [13,18], their occurrence in *H*. *vulgare* was observed for the first time. Their random localization in barley cells will not allow to classified them as dense bodies (DB), which are predominantly localized near nucleoli [18,19]. FISH together with traditional histochemical staining provides insight into the mechanisms of the formation of chromosome aberrations during genotoxicity studies. Such an approach has commonly been used in studies of structure and function of genomes, e.g. in *Allium cepa* [20] and *Brassica* species [21]. FISH and the analysis of the transcriptional activity of genes using traditional staining has not previously been applied in mutagenesis and environmental genotoxicity studies. Although analyses of the presence of rDNA loci in the micronuclei induced by mutagens has been previously performed [2,3], in this study their activity was analysed for the first time.

To conclude, this study is a first example of the application of the micronucleus test combined with analyses of the transcriptional activity of rRNA genes. Transcriptional activity is maintained in the micronuclei even taking into account the fact that they are eliminated during the next cell cycle. The analyses using this experimental model setup, including MH as a mutagen and barley as species, can be now extended to other abiotic stresses and plant species to study the response of plant cells to the environment.

## References

[1] Leitch IJ, Heslop-Harrison JS. Physical mapping of four sites of 5S rDNA sequences and one site of the alpha-amylase-2 gene in barley (*Hordeum vulgare*). Genome. 1993; 36: 517–523. 18470006 10.1139/g93-071

[2] Juchimiuk J, Hering B, Małuszyńska J. Multicolour FISH in an analysis of chromosome aberrations induced by N-nitroso-N-methylurea and maleic hydrazide in barley cells. J Appl Genet. 2007; 48(2): 99–106. 17495342 10.1007/BF03194666

[3] Juchimiuk-Kwasniewska J, Brodziak L, Małuszyńska J. FISH in analysis of gamma ray-induced micronuclei formation in barley. J Appl Genet. 2011; 52: 23–29. 10.1007/s13353-010-0017-x 21136232 PMC3026678

[4] Hubbel HR. Silver staining as an indicator of active ribosomal genes. Biotech Histochem. 1985; 60(5): 285–294. 10.3109/10520298509113926 2412317

[5] Rogers SO, Bendich AJ. Ribosomal RNA genes in plants–variability in copy number and in the intergenic spacer. Plant Mol Biol. 1987; 9: 509–520. 10.1007/BF00015882 24277137

[6] Hizume M, Sato S and Tanaka A. A highly reproducible method of nucleolus organizer regions staining in plants. Stain Technology. 1980; 55: 87–90. 6157230

[7] Maluszynska J, Heslop-Harrison JS. Localization of tandemly repeated DNA sequences in *Arabidopsis thaliana*. Plant J. 1991; 1(2):159–166.

[8] Unfriend I and Gruendler P. Nucleotide sequence of the 5.8S and 25S rRNA genes and of the internal transcribed spacers from *Arabidopsis thaliana*. Nucleic Acid Res. 1990; 18: 4011 2100998 10.1093/nar/18.13.4011 PMC331127

[9] Gabara B, Krajewska M, Stecka E. Calcium effect on number, dimension and activity of nucleoli in cortex cells of pea (*Pisum sativum* L.) roots after treatment with heavy metals. Plant Sci. 1995; 111: 153–161.

[10] Dolezel J, Cihalikova J, Zakchlenjuk OV. Sequential estimation of nuclear DNA and silver staining of nucleoli in plant cells. Stain Technol. 1989; 64(1): 9–13. 2472683 10.3109/10520298909108037

[11] Jabee F, Ansari MYK, Shaban D. Studies on the effect of maleic hydrazide on root tip Cells and Pollen Fertility in *Trigonella foenum-graecum* L. Turk J Botany. 2008; 32: 337–344.

[12] Guerrero F, De la Torre C, Garcia-Herdugo G. Control of nucleolar growth during interphase in higher plant meristem cells. Protoplasma. 1989; 152: 96–100.

[13] Barlow PW. Nucleolus-associated bodies (karyosomes) in dividing and differentiating plant cells. Protoplasma. 1983a; 115: 1–10.

[14] Małuszynska J, Hasterok R, Weiss H. rRNA genes–their distribution and activity in plants. W: Plant cytogenetics. Małuszynska J (Ed.) Wydawnictwo Uniwersytetu Śląskiego Katowice 1998 pp. 75–95.

[15] Jiang W, Liu D. Effects of Pb^2+^ on Root Growth, Cell Division and Nucleolus of *Zea* may*s* L. Bull Environ Contam Toxicol. 2000; 65(6): 786–793. 11080360 10.1007/s0012800191

[16] Qin R, Jiao Y, Zhang S, Jiang W, Liu D. Effects of aluminum on nucleoli in root tip cells and selected physiological and biochemical characters in *Allium cepa* var. agrogarum L. Plant Biol. 2010; 10: 225. 10.1186/1471-2229-10-225 PMC3017848 20964828

[17] Jiang Z, Zhang H, Qin R, Zou J, Wang J, Shi Q, Jiang W, Liu D. Effects of lead on the morphology and structure of the nucleolus in the root tip meristematic cells of *Allium cepa* L. Int J Mol Sci. 2014; 15: 13406–13423. 10.3390/ijms150813406 25089875 PMC4159802

[18] Williams LM, Jordan EG, Barlow PW. The ultrastructure of nuclear bodies in interphase plant cell nuclei. Protoplasma. 1983; 118: 95–103.

[19] Barlow PW. Changes in the frequency of two types of nuclear body during interphase of meristematic plant cells. Protoplasma. 1983b; 118: 104–113.

[20] Hasterok R, Małuszyńska J. Cytogenetic markers of *Brassica napus* L. chromosomes. J Appl Genet. 2000a; 41(1): 1–9.

[21] Hasterok R, Małuszyńska J. Different rRNA genes expression in primary and adventitious roots of *Allium cepa* L. Folia Histochem Cytobiol. 2000b; 8(4): 181–184. 11185723

